# Effect of 82% Lactic Acid in Treatment of Melasma

**DOI:** 10.1155/2014/407142

**Published:** 2014-07-17

**Authors:** Rashmi Singh, Sapna Goyal, Qazi Rais Ahmed, Narendra Gupta, Sujata Singh

**Affiliations:** ^1^Department of Pharmacology, Rohilkhand Medical College and Hospital, Bareilly 243006, India; ^2^Department of Skin and Venereology, Rohilkhand Medical College and Hospital, Bareilly 243006, India; ^3^Department of Physiology, Rohilkhand Medical College and Hospital, Bareilly 243006, India; ^4^Department of Pharmacology, Shri Ram Murti Smarak Institute of Medical Sciences, Bareilly 243202, India

## Abstract

Melasma is an acquired, chronic, and symmetrical hypermelanosis, characterized by brown patches of variable darkness on sun exposed areas of body. There are numerous modalities of treatment currently in use for this disease, of which the chemical peeling is very commonly used. Therefore, the present work was done to see the effect of 82% lactic acid peel in the treatment of melasma. A total number of 20 patients of either sex attending the OPD of dermatology department with clinically evident melasma were included in the study. 82% Lactic acid peel was applied on the face for 12 weeks in each patient. Patients were evaluated clinically and photographically at various intervals and in follow-up till 24 weeks. Assessment of patient satisfaction and side effects were also noted. All the subjects completed the study. Application of this peel for 12 weeks significantly decreased the melasma area severity index score and also melasma severity scale score. Patient and physician analogue scales also showed the improvement by the treatment. Regarding the adverse effects, burning sensation was the only side effect noted in our study. In conclusion, 82% lactic acid peel is well tolerated and can be used for the treatment of melasma.

## 1. Introduction

Melasma, also known as Chloasma or mask of pregnancy is an acquired, chronic, and symmetrical hypermelanosis, characterized by brown patches of variable darkness on sun exposed areas of body [1]. The exact causes of melasma are unknown. However, multiple factors are implicated in its etiopathogenesis which include genetic influence, exposure to UV radiation, pregnancy, and hormonal therapy (oral contraceptive pills and thyroid hormones). Other factors implicated are phototoxic drugs, anticonvulsant medication, and use of other cosmetics [2].

The disease usually presents as a single lesion to multiple patches located symmetrically on the face and occasionally “V” neck area [3]. There are three typical patterns of melasma which are clinically recognized: centrofacial, malar, and mandibular [4].

It may be classified according to natural history of lesions, according to clinical signs and symptoms and histologically on the basis of woodlamp.

Light microscopic findings of melasma include increased deposit of melanin in the epidermis and dermis or both when compared with adjacent normal skin and mild perivascular lymphohistiocytic infiltrates [5, 6]. Therefore the main aim of the treatment of melasma is to slow the proliferation of melanocytes and to inhibit formation of melanosomes as well as promotion of their degradation. These objectives can be achieved by inhibiting melanocytes activity, inhibiting melanin synthesis, removing melanin, and disrupting melanin granules contained within the melanosomes [7].

There are numerous modalities of treatment which are frequently used nowadays like sunscreens, topical depigmenting agents (like hydroquinone, azelaic acid, kojic acid, retinoids, and corticosteroids), chemical peels (superficial, medium, and deep), laser therapy, cryotherapy, and dermabrasion [8, 9].

Chemical peeling consists of application of one or more exfoliating agents on skin to obtain first destruction and then regeneration of part of the epidermis and/or dermis and it has become increasingly popular in the treatment of melasma [10]. According to Mark Rubin classification, these peels may be very superficial, superficial, medium, and deep depending on the depth of wound created by the peeling agents. Superficial and medium depth peels are safer for Indian patients [11].

Various chemicals used as peeling agents are alpha hydroxy acids (AHA) which include glycolic acid and lactic acid and beta hydroxy acids (BHA): salicylic acid, trichloroacetic acid, alpha keto acid, resorcinol, jessner's solution, retinoic acid, phenol, and so forth [12]. AHA are the most common agents used in current practice but the clinical use is limited to glycolic acid. Lactic acid, an AHA, is an old but innovative agent for peeling and it has similar activities like glycolic acid, but it has not been used as a common agent in the treatment of melasma [13]. So the purpose of the present work is to see the effect of 82% lactic acid (AHA) in the treatment of melasma.

## 2. Materials and Methods

### 2.1. Study Area

The present study was conducted in the Department of Dermatology, at Rohilkhand Medical College and Hospital, Bareilly, from October 2012 to March 2013 after approval of protocol by the local ethical committee.

### 2.2. Inclusion Criteria

A total of 20 patients of either sex, attending the outdoor patient department of dermatology department with clinically evident melasma and in good health with normal findings in medical history and physical examination were included in the study.

The realistic outcome of the treatment was discussed with each and every patient and an informed written consent was taken before inclusion into the study and if the patient was below 18 years, consent was taken from the parents/guardian.

### 2.3. Exclusion Criteria

Pregnant females, one-year postdelivery or lactating females, females on oral contraceptive pills, patients with preexisting skin disease like inflammatory dermatoses, psoriasis, atopic dermatitis, patients with history of drugs or with photosensitizing potential or active bacterial, viral, and fungal infections, and patients with keloid tendency were excluded from the study. History of chemical peeling or any other surgical procedures on the face during last six months, patients with psychological problems which may lead to noncompliance, or patients who could not keep themselves away from sun exposure especially during the course of chemical peeling were also excluded from the study.

### 2.4. History and Examination

Detailed demographic data, clinical history including the onset, duration, site, and progression, precipitating factors, seasonal variation, treatment received, any prolonged drug intake, obstetric and menstrual history, history of melasma onset in pregnancy and its aggravation with further pregnancies, family history, and so forth were taken from all the patients and recorded in a preset proforma.

A complete examination including general physical and dermatological examinations was carried out. Detailed examination of the face under good illumination was conducted to assess total number of lesions, size of the lesion, clinical type, homogeneity, and colour of lesion. All the patients in the study were classified according to their Fitzpatrick skin phototypes.

### 2.5. Materials

Materials used were 82% lactic acid (Percos Company), cotton wool applicator, spirit, petroleum jelly, cotton gauze, graduated dispensing cup, acetone, digital camera, gloves, and fan for cooling.

### 2.6. Prepeeling Procedure

For the peeling procedure, prepeeling priming was done in all the patients 2 weeks prior to procedure by applying broad spectrum sunscreens and 4% hydroquinone on the lesion area for 30 minutes to overnight depending on the tolerability of hydroquinone.

At the first visit, a test peel was done on the postauricular area of 1 cm^2^ after cleaning with acetone followed by spirit and then 82% lactic acid. The patients were reviewed after one week for any type of erythema, scaling, and crusting in the test peel area. If the patients tolerated the peel well, they were taken up for full face peel. The peel was applied to the faces serially at 2 weekly intervals at 0, 2, 4, 6, 8, 10, and 12 weeks after test peel of 1 week.

### 2.7. Peeling Procedure

After cleansing and degreasing the face, the patients were lying in semireclining position with eyes closed comfortably on their back. Degreasing was done by scrubbing with a cotton gauze soaked with spirit followed by one soaked with acetone. Sensitive areas of the face like lips and nasolabial folds were protected with a thin layer of petroleum jelly. After that peeling was started by applying 82% of lactic acid over the face with cotton wool applicator dipped in the required solution with smooth strokes to the affected areas. A contact time of 2 minutes at 0 weeks and 5 minutes at 2 weeks was given and this contact period was subsequently increased by 5-5 minutes at 4, 6, 8, 10, and 12 weeks. Time at which untoward side effects appeared was noted and the duration of application of chemical was kept constant at that for next further visits for that patient.

For lactic acid peel, extreme burning or erythema or epidermolysis seen as grayish white appearance of epidermis was considered as the end point of the peeling session. Then termination of peeling was done by cleaning the face with cold water and drying but without rubbing. Patients were advised to avoid washing face with soap at least for next 24 hours, avoid sun exposure, and apply sunscreen of SPF 50 at daytime after peeling. Side effects if any noticed during the study were recorded in the preset proforma.

## 3. Clinical Evaluation

For the treatment outcome, clinical evaluation of melasma severity was done by melasma area severity index (MASI) and melasma severity scale (MSS). Both were calculated for every session of treatment, that is, at 2, 4, 6, 8, 10, and 12 weeks. Subjective assessment was evaluated on visual analogue scale (VAS), by scoring between 0 and 5. After treatment, patients were followed up monthly for three months to note any recurrence and to record the side effects if any. Evaluations were done at 16, 20, and 24 weeks using MASI, MSS, and VAS.

Statistical analysis was done by applying paired* t*-test, using Microsoft excel 2007 software as applicable and* P* values of less than 0.05 were considered significant.

## 4. Results

**Figure 1:**
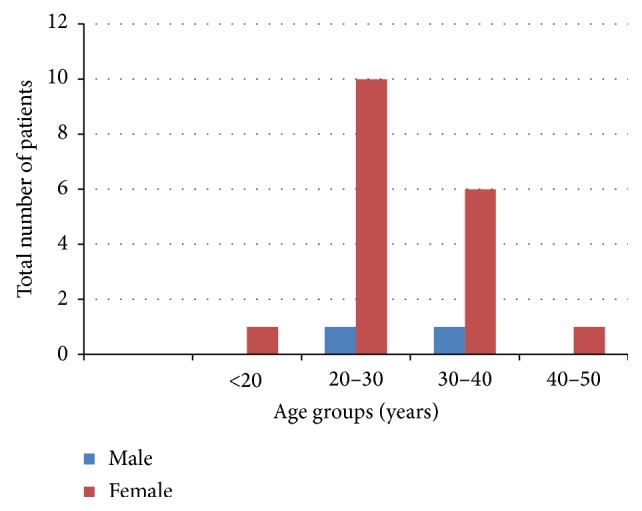
Distribution of patients according to sex and age groups.

Regarding the demographic data, a total of 20 patients were included in the study among which females comprised 90% (*n* = 18) of the study population with M : F ratio of 1 : 9. The maximum number of female patients belongs to age group of 20–30 years (Figure 1). The mean age of melasma patients in our study was 28.6 ± 5.01 years; mean age of the females was 28.44 ± 5.26; and that of males was 30 ± 1.414 years.

Majority of the patients 75% (*n* = 15) were married while others 25% (*n* = 5) were single. Similarly, 65% (*n* = 13) of the patients were educated while 35% (*n* = 7) were uneducated. 25% (*n* = 5) of patients had positive family history of melasma in the present study.

**Figure 2:**
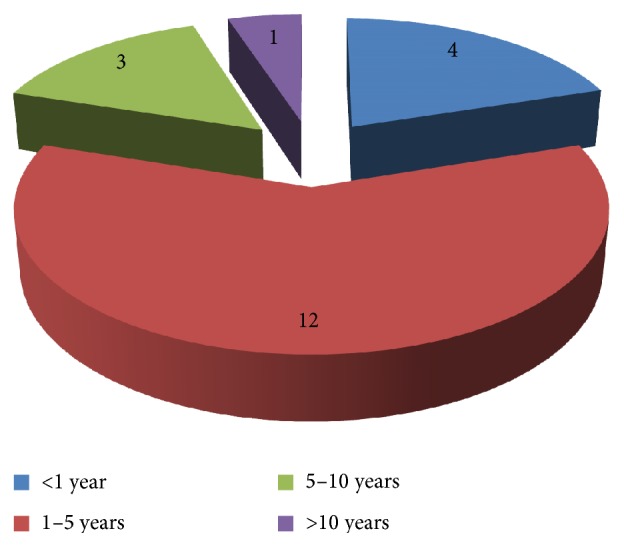
Distribution of patients according to duration of disease.

Duration of the disease ranged from 4 months to 12 years with mean duration being 3.825 years. Most (60% (*n* = 12)) of the patients in our study had onset of melasma in preceding 1–5 years (Figure 2).

In our study, 35% (*n* = 7) of patients reported pregnancy as a precipitating factor and 20% (*n* = 4) reported sunlight as a precipitating factor while 45% (*n* = 9) had no specific factor.

All the patients belonged to Fitzpatrick skin phototypes IV and V. 65% (*n* = 13) of total patients belonged to skin phototype V and 35% (*n* = 7) to type IV and regarding the pattern of lesions in our study, malar pattern was the most common, that is, 60%, and the others had centrofacial type (40%). No one had mandibular type of melasma.

50% of the subjects have used some topical treatment in the past with no improvement while remaining half never received any treatment for melasma.

**Figure 3:**
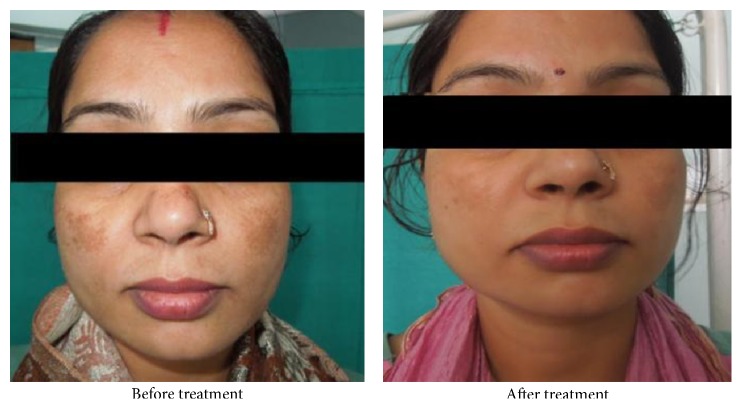


In our study, patients treated with 82% lactic acid showed reduction in posttreatment MASI score when compared with baseline. Mean MASI score after 12 weeks of treatment was 1.865 while that at baseline was 2.885 representing a decrease of 1.02 and a % improvement of 35.36% at 12 weeks. In follow-up period the MASI score decreased to 1.86 at 16 weeks and to 1.855 at 20 and 24 weeks showing the improvement of 35.7% at 24 weeks (Tables 1 and 2 and Figure 3).

Regarding the melasma severity scale, at 0 weeks, maximum number (65%) of patients belonged to grade 2 melasma, followed by grade 1 and grade 3. The order changed to grade 1 (80% patients) followed by grade 2 and grade 3 after 12 weeks of treatment as a larger number of patients shifted from grades 2 to 1 and from grades 3 to 1 by the treatment and the results were maintained in follow-up period also (Table 3).

On physician VAS, 55% of patients showed VAS1 (0–25% improvement) while only 30% of patients fell into VAS2 (25–50%) after 12 weeks of treatment which were maintained in follow-up period also (Table 4).

On patient VAS, 70% of patients showed improvement between 0 and 25% (VAS 1) at 12 weeks while maximum improvement of 25–50% (VAS 2) was seen in 30% of patients at 2, 4, 6, and 8 weeks which was reduced to 25% of patients at 10 weeks and thereafter to 15% at 12 weeks and also in follow-up period (Table 5).

## 5. Discussion

Melasma is a very common problem in dark skinned people in which there is an acquired increased pigmentation of the skin [3, 14]. It is a commonly seen entity in clinical practice that affects women of reproductive age group, during pregnancy in particular [15].

There are various modalities of treatment of melasma. One of them is the bleaching agents such as hydroquinone, but unfortunately relapse rate is usually high after therapy, that may be attributed to continuous exposure to triggering agents like sunlight, which is the main exacerbating factor.

Superficial and medium depth chemical peels have been employed with variable success in the treatment of melasma but this procedure may have some potentially undesirable side effects and tolerance to this procedure may vary from person to person [16–18].

Regarding the demographic data, the mean age of melasma patients in our study was 28.6 ± 5.01 years, which was in accordance with other studies done by various authors such as Sharquie et al., Sarvjot et al., and Khunger et al. [13, 19, 20] but one of the studies reported from Singapore done by Goh and Dlova found that the mean age was 42.3 years [21]. In our study mean age of the females was 28.44 ± 5.26 and that of males was 30 ± 1.414 years. Most of the females belonged to age group of 20–30 years while there were only two males one belonging to 20–39 years of age group while the other one was in age group of 30–40 years.

Melasma is more common in women. We found the same in our study where females comprised 90% of the study population. This was supported by a study done in India by Sarkar et al. [22, 23]. Another study by Sarvjot et al. also showed the female predominance with M : F ratio of 1 : 3 [20]. We found about 10% involvement of men in our study similar to studies by Katsambas et al. and Vázquez et al. [24, 25]. Female preponderance noted in our study can be explained by the high incidence of melasma in women and also pregnancy, oral contraceptive pills, and estrogen-progesterone therapies implicated as various factors in the etiopathogenesis of this condition. Moreover, females are more conscious of the cosmetic disfigurement caused by melasma which may be one of the important reasons for more females presenting to the outpatient department for treatment.

A positive family history was observed in 25% (*n* = 5) of patients, in the present study, which was in accordance with various reported studies and most of these were in the first degree relatives [13, 25–27]. However another study by Goh and Dlova [21] observed positive family history only in 10.2% (*n* = 21) patients [21]. The presence of a family history suggests that genetic factors may play a role in the pathogenesis of melasma. However, larger studies are required to determine the role of genetic inheritance.

In our study duration of the disease ranged from 4 months to 12 years with mean duration being 3.825 years which was in accordance with the study of Sharquie et al. and İlknur et al. but not with Goh and Dlova [21] where onset of disease in most patients was more than 5 years [13, 21, 27]. Most (60% (*n* = 12) of the patients in our study had onset of melasma in preceding 1–5 yrs while only 5% (*n* = 1) had long standing disease of more than 10 years. This can be attributed to increased aesthetic awareness amongst the people nowadays.

Female hormones, sunlight, and genetic predisposition are three main factors in the development of melasma. In this study only 35% (*n* = 7) of the patients noted pregnancy as a precipitating and aggravating factor, while 20% (*n* = 4) of the patients noted sunlight as a precipitating and aggravating factor. These figures are opposite to those reported earlier. Few other studies have also reported a minimum relation with either pregnancy or oral contraceptives [28, 29] while other few studies confirmed the association of cosmetics and melasma such as Grime [2].

In our study all the patients belonged to Fitzpatrick skin phototypes IV and V. 65% of total patients (*n* = 13) belonged to skin type V and 35% (*n* = 7) to 4th skin type which was in accordance with Sharquie et al. [13].

According to the distribution of the lesions three clinical patterns were recognized in our study and among these, malar type was the most common, like other studies from Singapore and South India [21, 30]. However other studies from India and abroad observed centrofacial distribution as the most common type [8, 30]. This variation of results might be due to environmental or regional differences.

In the treatment history 50% of our study subjects have used multiple topical treatments from outside with no improvement. However our outcome measures were not affected by the previous modality because a washout period of 4 weeks was used in our study, where patients were instructed not to use any of the previously used medicines.

Conventional treatment for melasma includes elimination of any possible causative factors along with other measures. Bleaching agents and chemical peels are the most frequently used methods in the treatment of melisma.

In this study, we have seen the effect of 82% lactic acid in patients of melasma which showed reduction in posttreatment MASI score when compared with baseline. The decrease in MASI score was statistically significant (*P* < 0.05) at 12 weeks and all follow-ups. There was 35.7% of total improvement at final visit (24 weeks). Mean MASI score at baseline was 2.885 which decreased to 1.865 at 12 weeks, 1.86 at 16 weeks, and finally to 1.855 at follow-up of 20 and 24 weeks while study done by Sharquie et al. with 92% LA demonstrated a mean decrease of 7.76 in MASI representing 79.34% improvement [13]. Another study done by the same author again demonstrated a mean decrease of 7.97 in MASI representing 56.72% improvement [31]. The difference in results may be because of the profound difference in skin types as in our study all patients were of darker skin types belonging mostly to Fitzpatrick skin type V while in the previous studies patients belong to Iraqi population with fairer skin types of IV having epidermal melasma and best therapeutic results are normally achieved in epidermal melasma [32]. Similarly past history of melasma was little bit long (4 months to 12 years) in our study as compared to above mention study (2 to 10 years). One more but not so important factor of strength of LA can also contribute to difference in results (82% versus 92%).

The other existing studies of AHA have been done in melasma with glycolic acid (GA) peels. One of the studies by Javaheri et al. on Indian patients with melasma strongly supported our study by showing 35.4% improvement in MASI with 50% GA peel [33].

However other studies by Sarkar et al. and Lawrence et al. showed 77.99% and 63% of improvement by using 30 to 40% and 70% of GA, respectively [34, 35]. This difference in results can be attributed to the use of concomitant therapy where they used modified Kligman formula and 0.05% tretinoin along with 4% hydroquinone in between the peels. Thus, the use of concomitant therapy might have led to increased improvement with GA peels, which was not used in our study.

Melasma severity scale also showed improvement by the treatment. At 0 weeks, maximum number of patients (65%) reported grade 2 melasma which decreased to 15% after 12 weeks of treatment as more number of patients shifted to grade 1 in which 25% patients were there at baseline that rises to 80% at 12 weeks. Statistically significant difference was seen in grade 2 patients at 8, 10, and 12 weeks (*P* value < 0.05) and the results remained statistically significant (*P* value < 0.05) at each follow-up till 24 weeks.

On physician VAS, 55% of patients showed VAS 1 (0–25% improvement) while only 30% patients fell into VAS 2 (25–50%) after 12 weeks of treatment which were maintained at till last follow-up. On patient VAS, 70% of patients showed improvement between 0 and 25% (VAS 1) at 12 weeks while maximum improvement of 25–50% (VAS 2) was seen in 30% of patients at 2, 4, 6, and 8 weeks which was reduced to 25% of patients at 10 weeks and thereafter to 15% at 10 and 12 weeks and also at various follow-ups.

In our study burning sensation was the only side effect noted with 82% lactic acid treatment. Sharquie et al. did not observe any side effect and considered LA as safe and well tolerated in the treatment of melasma [13, 31]. But the side effects noted by the use of glycolic acid include erythema, superficial desquamation, burning, and vesiculation as given by Khunger et al. [19].

Recurrence after treatment is one of the most difficult aspects about treatment of melasma. We did not notice any recurrence in our study in 3-month follow-up period and improvement was maintained which was significant.

## 6. Conclusion

This study showed that application of 82% lactic acid peel decreased the MASI score which was statistically significant. Melasma severity scale score also showed significant difference in grade 2 patients. Patient and physician analogue scales also showed the improvement by lactic acid treatment. Besides these no side effects were noted in our study except the burning sensation. Therefore we conclude that 82% lactic acid peel is well tolerated and safe agent that can be used for the treatment of melasma but more studies on Indian patients using larger sample sizes are needed to validate our results.

## Figures and Tables

**Table 1:** Mean MASI score at various intervals after application of lactic acid.

Duration	0 weeks	2 weeks	4 weeks	6 weeks	8 weeks	10 weeks	12 weeks	16 weeks	20 weeks	24 weeks
MASI score	2.885	2.715	2.37	2.125	1.995	1.885	1.865	1.86	1.855	1.855

**Table 2:** Change in MASI score and percentage improvement by lactic acid.

Time interval	Improvement	% improvement	*P* value
0–2 weeks	0.170	5.893	0.752
0–4 weeks	0.515	17.851	0.331
0–6 weeks	0.760	26.343	0.151
0–8 weeks	0.890	30.849	0.079
0–10 weeks	1.000	34.662	0.056
0–12 weeks	1.020∗	35.355∗	0.049∗
0–16 weeks	1.025∗	35.529∗	0.048∗
0–20 weeks	1.030∗	35.702∗	0.047∗
0–24 weeks	1.030∗	35.702∗	0.047∗

*P* value compared to baseline (0 weeks), ^*^ *P* value < 0.05.

**Table 3:** Total number of patients in grades I, II, and III of melisma severity scale at different durations.

		0 weeks	2 weeks	4 weeks	6 weeks	8 weeks	10 weeks	12 weeks	16 weeks	20 weeks	24 weeks
Grade 1	Number of pt	5	6	10	15∗	16∗	16∗	16∗	16∗	16∗	16∗
	% of pt	25%	30%	50%	75%	80%	80%	80%	80%	80%	80%
	*P* value		0.683	0.114	0.0098	0.006	0.006	0.006	0.006	0.006	0.006

Grade 2	Number of pt	13	13	9	4∗	3∗	3∗	3∗	3∗	3∗	3∗
	% of pt	65%	65%	45%	20%	15%	15%	15%	15%	15%	15%
	*P* value		1	0.267	0.013	0.0055	0.0055	0.0055	0.0055	0.0055	0.0055

Grade 3	Number of pt	2	1	1	1	1	1	1	1	1	1
	% of pt	10%	5%	5%	5%	5%	5%	5%	5%	5%	5%
	*P* value		0.479	0.479	0.479	0.479	0.479	0.479	0.479	0.479	0.479

*P* value refers to change in the number of patients at different intervals compared to “0” week, ^*^ *P* value < 0.05.

**Table 4:** Physician visual analogue scale with lactic acid treatment at different durations.

	0 weeks	2 weeks	4 weeks	6 weeks	8 weeks	10 weeks	12 weeks	16 weeks	20 weeks	24 weeks
VAS 0	12	9	8	6	4	4	3	3	3	3
No improvement	60%	45%	40%	30%	20%	20%	15%	15%	15%	15%

VAS 1	6	8	9	10	11	11	11	11	11	11
0–25% improvement	30%	40%	45%	50%	55%	55%	55%	55%	55%	55%

VAS 2	2	3	3	4	5	5	6	6	6	6
25–50% improvement	10%	15%	15%	20%	25%	25%	30%	30%	30%	30%

**Table 5:** Patients visual analogue scale with lactic acid treatment at different durations.

	0 weeks	2 weeks	4 weeks	6 weeks	8 weeks	10 weeks	12 weeks	16 weeks	20 weeks	24 weeks
VAS 0	6	3	2	2	2	3	3	3	3	3
No improvement	30%	15%	10%	10%	10%	15%	15%	15%	15%	15%

VAS 1	10	11	12	12	12	12	14	14	14	14
0–25% improvement	50%	55%	60%	60%	60%	60%	70%	70%	70%	70%

VAS 2	4	6	6	6	6	5	3	3	3	3
25–50% improvement	20%	30%	30%	30%	30%	25%	15%	15%	15%	15%
